# Oral bisphosphonate-related osteonecrosis of the jaws: Clinical characteristics of a series of 20 cases in Spain

**DOI:** 10.4317/medoral.18041

**Published:** 2012-05-01

**Authors:** Márcio Diniz-Freitas, José L. López-Cedrún, Jacinto Fernández-Sanromán, Abel García-García, Javier Fernández-Feijoo, Pedro Diz-Dios

**Affiliations:** 1 Oral Medicine and Oral surgery Units, School of Medicine and Dentistry, University of Santiago de Compostela, Santiago de Compostela, Spain; 2 Department of Oral and Maxillofacial Surgery, A Coruña University Hospital Complex (CHUAC), A Coruña, Spain; 3 Department of Oral and Maxillofacial Surgery, Policlínico Vigo S.A. (POVISA), Vigo, Spain; 4 Department of Oral and Maxillofacial Surgery, Santiago de Compostela University Hospital Complex (CHUS), Santiago de Compostela, Spain; 5 Grupo de Investigación en Odontología Médico-Quirúrgica (OMEQUI), School of Medicine and Dentistry, University of Santiago de Compostela, Santiago de Compostela, Spain

## Abstract

Objective: The objective of this study was to define the clinical characteristics of osteonecrosis of the jaws (ONJ) induced by oral bisphosphonates in a series of patients from a circumscribed area in northwest Spain. Study Design:A retrospective multicentre study was undertaken in 3 hospitals in an area with a radius less than 100 km in the Autonomous Community of Galicia (Spain). The medical records were reviewed and an oral examination was performed of patients diagnosed with oral bisphosphonate-related ONJ in the previous 3 years. Results: We detected 20 cases of ONJ (24 lesions) related to oral bisphosphonates (alendronate [16 patients] and ibandronate [4 patients]), which were mainly administered as treatment for osteoporosis (17 patients). The mean interval between initiation of treatment and confirmation of a diagnosis of ONJ was 66±43 months (range, 6-132 months); in 7 patients (35%) the interval was less than 36 months. The past history revealed hypertension in 13 cases (65%) and diabetes in 4 (20%); 7 patients (35%) were on corticosteroid treatment. Oral surgery had been previously performed in 13 patients (65%) and the remaining 7 patients (35%) had removable dental prostheses. The lesions most frequently affected the posterior mandible (62.5%). The majority of the lesions (75%) were classified as stage 2, although lesions were identified in all established clinical stages (including 2 stage 0 lesions). Conclusion: In conclusion, in the present series, ONJ induced by oral bisphosphonates typically develops in women around 70 years of age, taking alendronate, that underwent oral surgery. Most lesions are located in the posterior mandible and are classified as stage 2 at diagnosis. Some patients presented no known risk factors, suggesting that there may be risk factors still to be identified. There are well-defined patterns of clinical presentation that can facilitate early diagnosis of ONJ.

** Key words:**Oral bisphosphonates, osteonecrosis of the jaws, alendronate.

## Introduction

Osteoporosis is the most common metabolic bone disease and affects more than 200 million individuals worldwide (1); in Spain alone it affects more than 3 million individuals. As a result, there has been a progressive increase in recent years in the prescription of drugs to combat the disease, particularly oral bisphosphonates (2). The oral bisphosphonates most widely used for the treatment of osteopenia and osteoporosis are alendronate, risedronate, and ibandronate (3). Their efficacy derives mainly from their ability to inhibit bone resorption, which results in a significant reduction in the prevalence of vertebral and nonvertebral fractures in postmenopausal women with osteoporosis.

New adverse effects of the prolonged administration of bisphosphonates have been detected in recent years; one of these, particularly important due to its associated morbidity, is osteonecrosis of the jaws (ONJ). This is defined as an area of persistent bone exposure in patients treated with bisphosphonates but who have not received radiotherapy (4). The relationship between intravenous bisphosphonates and ONJ is based on firm epidemiological evidence, however, ONJ attributable to the use of oral bisphosphonates has been a subject of considerable controversy, although recent studies have provided evidence of this association (5). As patients with osteoporosis require prolonged treatment, there has been an increase in the number of cases of ONJ related to the use of oral bisphosphonates, and in a recently published retrospective multicentre study it was suggested that the relative frequency of ONJ in patients with osteoporosis treated with oral bisphosphonates was higher than previously estimated (6).

The objective of this study was to define the clinical characteristics of a series of patients with ONJ induced by oral bisphosphonates in a circumscribed area in northwest Spain.

## Material and Methods

A retrospective multicentre study was undertaken in 3 hospitals in an area of less than 100 km in radius in the Autonomous Community of Galicia (Spain): Santiago de Compostela University Hospital Complex (CHUS), A Coruña University Hospital Complex (CHUAC), and Policlínico Vigo S.A. (POVISA) in Vigo. We reviewed the medical records of all patients who had been diagnosed with ONJ related to the use of oral bisphosphonates during the period from May 2008 through April 2011 in any of the 3 involved hospitals. The inclusion criteria were those established by the American Association of Oral and Maxillofacial Surgeons for the diagnosis of bisphosphonate-related ONJ (4): bone exposure in the maxillofacial region that persists for more than 8 weeks in patients treated with bisphosphonates (currently or previously) and who have not received radiation therapy to that anatomical region. In 2009 these directives were updated to include patients with stage 0 disease (7). Other possible causes of ONJ were excluded on the basis of the medical history and, when necessary, additional tests (panoramic radiographs, computed tomography, histopathology examination, and microbiology cultures) were performed in order to establish the definitive diagnosis. All identified patients were recalled for a follow-up visit at which details missing from the medical history that could be relevant for ONJ development were gathered (e.g. tobacco use) and a detailed examination of the oral cavity was performed.

The following variables were analyzed: sex, age, type of bisphosphonate, duration of treatment, comorbid conditions and coadjuvant medication, smoking, previous dental procedures, clinical presentation, site affected, size and clinical stage of the lesions (5). Informed consent was obtained from all patients who participated in the study. The study was reviewed by the Institutional Review Board (Galician Health Service [SERGAS]), but because of its retrospective nature it was exempt from formal approval.

## Results

Demographic and clinical characteristics of patients with oral bisphosphonates related osteonecrosis of the jaws is shown in ( Table 1). Twenty caucasian patients (19 women and 1 man) were identified with a total of 24 lesions that satisfied the inclusion criteria. All patients but 2 were born in Galicia (the remaining 2 patients lived in this area for more than 20 years). Seventeen patients were taking oral bisphosphonates for the treatment of osteoporosis and 3 for the prevention of osteoporosis secondary to glucocorticoid therapy for rheumatoid arthritis. The mean age was 71.2±7.5 years (range, 53-82 years). The oral bisphosphonates administered were alendronate (16 patients, 80%) and ibandronate (4 patients, 20%). The mean interval between the initiation of treatment and confirmation of a diagnosis of ONJ was 66±43 months (range, 6-132 months; median, 55 months). In 7 (35%) patients the interval was less than 36 months. With regard to concomitant diseases and medication, 13 (65%) patients were hypertensive, 4 (20%) were diabetic, and 7 (35%) were taking corticosteroids.

**Table 1:**
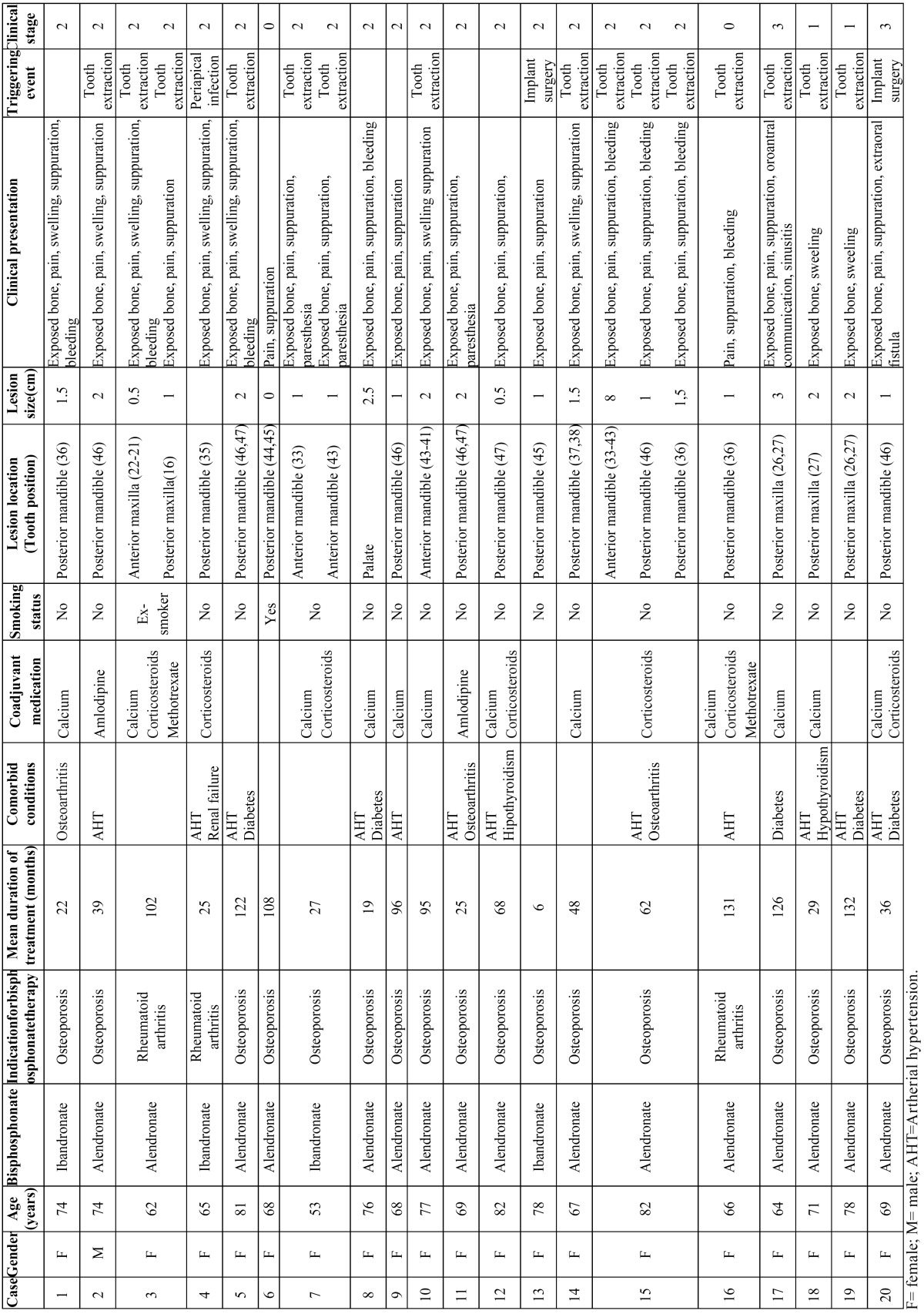
Demographic and clinical characteristics of patients with oral bisphosphonates related osteonecrosis of the jaws(n= 20)

A history of oral surgery in the 12 months prior to the appearance of the lesions of osteonecrosis was detected in 13 (65%) patients. Eleven of those patients had undergone tooth extractions and 2 had endosseous implants placed during treatment with oral bisphosphonates. The remaining 7 (35%) patients had not undergone surgical dental procedures in the 12 months prior to the diagnosis of ONJ, although all had removable dental prostheses.

Three (15%) patients presented more than 1 ONJ lesion at the time of diagnosis: 2 lesions in 2 patients and 3 lesions in 1 patient. The majority of lesions (62,5%) were in the mandible, particularly affecting the region of the molars and premolars. The most common signs and symptoms were bone exposure, pain, suppuration, and inflammation ( Table 1).

Orosinusal communication and sinustis were observed in 1 patient, and the presence of an extraoral fistula in another; these 2 lesions (8.3% of all the lesions observed) were classified as stage 3. The majority of the lesions (18/24 [75%]) were classified as stage 2, with exposure of necrotic bone and pain and/or signs of infection. Two lesions were classified as stage 1, with exposure of necrotic bone without pain or signs of infection. The 2 remaining lesions were classified as stage 0, without bone exposure but with nonspecific signs and symptoms of ONJ.

## Discussion

Although the description of bisphosphonate-related ONJ is relatively recent (8), the condition has become important in the field of dentistry due to the increased number of reported cases and the difficulty of its therapeutic management, even in patients treated with oral bisphosphonates (6). In a open PubMed-based search (until April 2011) we found 14 case series with at least 10 patients with oral bisphosphonate-related ONJ; added to the present series, we thus found a total of 310 patients (6,9,21) ( Table 2- Table 2 (continue)). The first European series of ONJ was published in Spain by Bagán et al. (22) and described 10 oncology patients with ONJ after treatment with intravenous bisphosphonates. The present study describes the clinical presentation and risk factors in a series of nononcology patients with ONJ related to treatment with oral bisphosphonates. After reviewing the literature, we believe that this is the largest case series described in Spain, and it´s among the top-ten of the largest case series published worldwide including oral bisphosphonate-related osteonecrosis patients detected in a single city or a delimited geographical region. The main limitation of this study is that no information was gathered regarding the treatment of the lesions and their clinical course; our data do not therefore contribute to this area of clinical relevance. Other important bias is that clinical characteristics of patients treated in other outpatient institutions or by other specialists in this geographical area, remain unknown. Moreover, as clinical stage 0 was defined in 2009 directives and the cases were collected from May 2008, stage 0 patients may be underestimated. One of the strengths of the study is that it was carried out in a limited geographical area, reducing the possibility of selection bias and reducing the potential influence of ethnic, social, cultural and genetic factors.

**Table 2:**
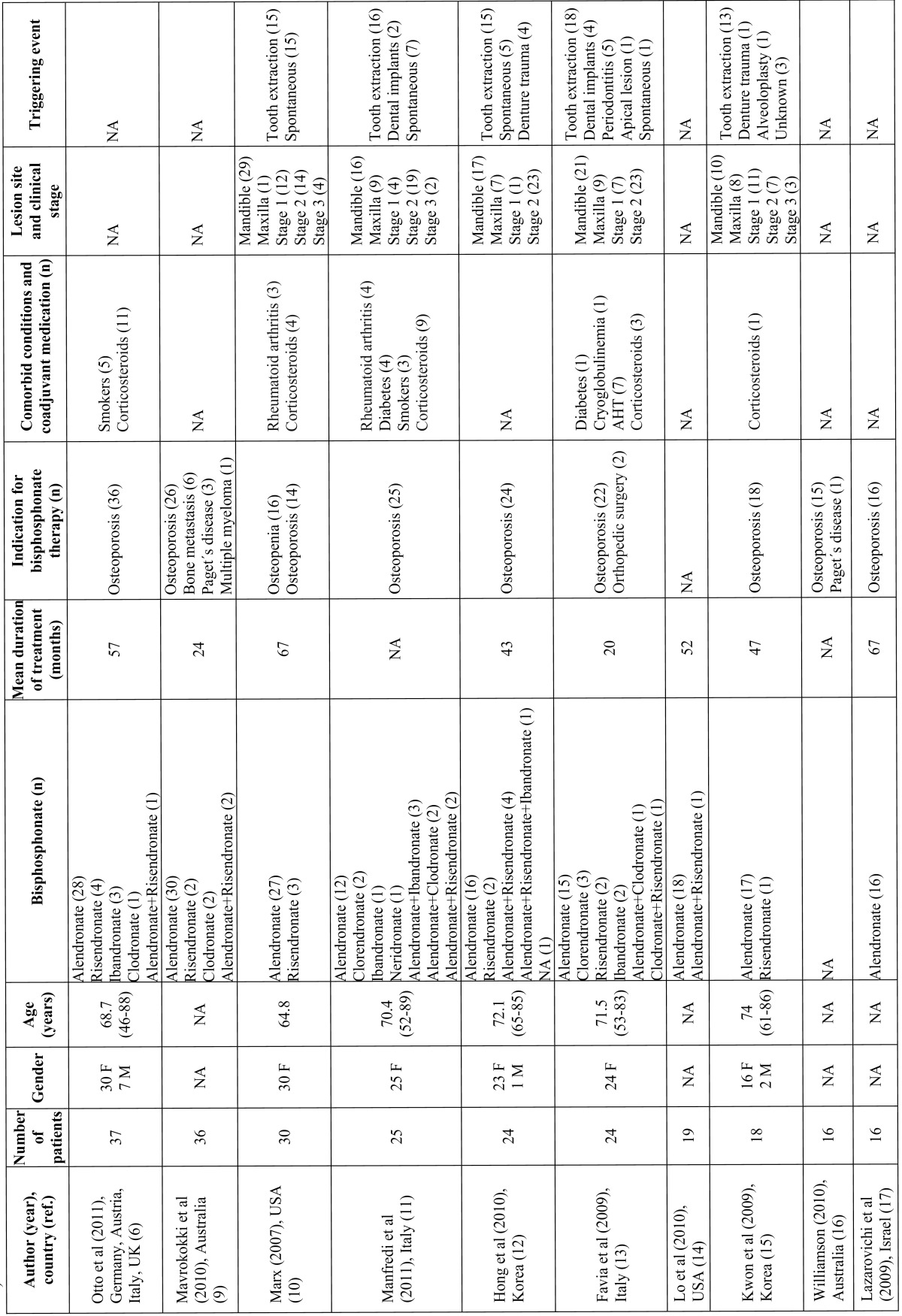
Selected characteristics of the main published series on oral bisphosphonates related osteonecrosis of the jaws (including at least 10 patients and with available clinicopathological information).

**Table 2 (continue):**
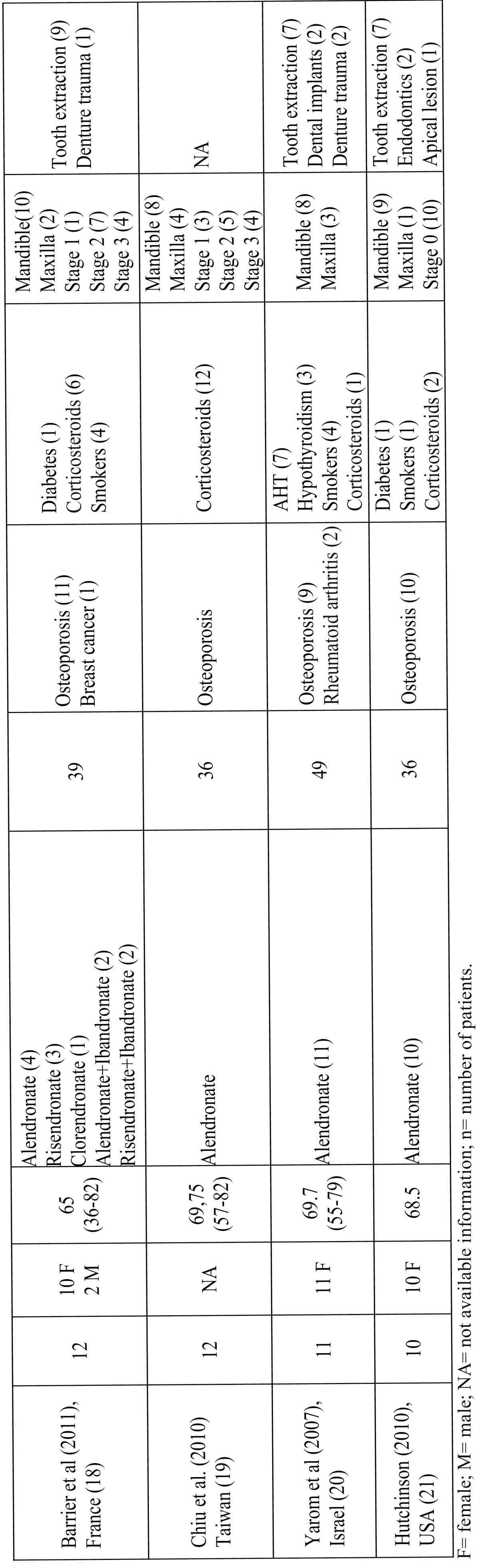
Continue table 2.

As in other studies, alendronate was the drug most commonly used for the treatment of osteoporosis in our patients. It is estimated that the incidence of ONJ in patients treated with oral bisphosphonates is approximately 0.7 cases per 100000 population per year (23), although some authors consider the true figure to be higher, and it has been suggested that the increase in the long-term use of this medication will lead to a rise in the incidence of ONJ.

One of the main risk factors for the onset of oral bisphosphonate-related ONJ discussed in the literature is the duration of treatment. In a review of the series published up to the year 2009, it was stated that the mean duration of treatment with oral bisphosphonates prior to the appearance of lesions of ONJ was 4.6 years (24). In our series, the mean time to presentation was slightly longer than the figure reported in the literature, although it was within the ranges observed. However, a considerable number of patients (35%) in our series developed ONJ after less than 3 years of treatment with oral bisphosphonates, a situation that has already been described in the literature (25). The onset of ONJ thus appears to be determined not only by the duration of the treatment but also by other risk factors, which may include local injury, systemic diseases, concomitant medication, and genetic predisposition. Twelve (68%) of the 19 women included in our series had systemic hypertension and were receiving antihypertensive medication; this percentage is higher than that of Spanish women with hypertension in the general population in the age range 65 to 74 years (55%). However, this could be due to the combined influence of osteoporosis and hypertension, based on common pathophysiologic mechanisms, as the changes in hormone metabolism associated with the menopause may be implicated in the development of both conditions (26). Another possible risk factor for ONJ is diabetes, particularly in oncology patients treated with intravenous bisphosphonates (27). In the present series we found that 20% of patients had diabetes, a higher prevalence than in the adult population from the rest of Spain (10-15%) (28). However, taking into account that diabetes is a risk factor for osteoporosis (29), that the frequency of diabetic patients in other series is lower, the retrospective nature of the present study, and the limited number of cases, the importance of diabetes as a risk factor for ONJ in patients treated with oral bisphosphonates will need to be confirmed in future studies.

Although there is no scientifically demonstrated link between bisphosphonate-related ONJ and rheumatoid arthritis, a number of hypotheses have recently been proposed that suggest that rheumatoid arthritis could be a risk factor for bisphosphonate-related ONJ. The mechanisms that have been discussed as possible links between these disorders include the actions of certain inflammatory mediators and the effects of drugs commonly used in the treatment of rheumatoid arthritis, particularly the steroids and methotrexate (MTX), which appear to play a relevant role in the onset of bisphosphonate-related ONJ (30). The drug group most commonly discussed in the literature are the corticosteroids. Experimental studies in rats (31) have shown that the combined administration of bisphosphonates and corticosteroids produces changes in the hard and soft tissues that are similar to those found in patients who develop oral lesions of bisphosphonate-related ONJ. Recently, Chiu et al. (19) published a series of 12 patients with clinicopathologically proved oral bisphosphonate-related ONJ; all the patients were concurrently administered long-term corticosteroids; in these patientes ONJ lesions were severe and treatment outcome was unpredictable.In our series, a total of 7 patients were receiving corticosteroids, 3 of whom had been diagnosed with rheumatoid arthritis.

Yarom et al. (20) suggested a possible relationship between ONJ and smoking. All patients in their series were smokers. In contrast, in a later study, Favia et al. (13) presented a series of 24 nonsmokers with ONJ related to oral bisphosphonates; our results are consistent with that report, as only 2 of our patients were smokers and 1 an ex-smoker. Although there have been reports of spontaneous ONJ, those cases are usually associated with local injury, almost always related to oral surgery, particularly to tooth extractions and the insertion of dental implants (19). It has been shown that bisphosphonates alter osteoclast function, leading to a delay in the initial phase of healing of the socket after tooth extraction; this could favor secondary infection and the onset of ONJ (32). In our series, we found a considerable number of cases, higher than in other series (13,15,18,20) in which the onset of ONJ was not associated with previous surgical dental procedures. Bisphosphonates have recently been shown to have an inhibitory effect on the proliferation of keratinocytes in the oral mucosa (33), and it has been suggested that poorly fitting removable dental prostheses could injure the underlying mucosa, increasing the risk of developing ONJ in the affected bone (34).

The clinical presentation of ONJ in our patients, with regard both to the site and to the clinical stage of the lesions, was similar to that reported in other studies. The predilection for posterior areas of the mandible has also been described in other series, as has the predominance of patients with stage 2 disease (exposure of necrotic bone with pain and/or signs of infection) (10-13,18).

The oral bisphosphonates are the most widely used drugs for the management of osteoporosis, with millions of users worldwide. In Spain, these drugs are among the top 10 therapeutic subgroups in terms of number of prescriptions and health-related costs in the Spanish national health system. As a result, it may be predicted that the number of cases of oral bisphosphonate-related ONJ will increase in the coming years, and there is an urgent need to substantiate epidemiological characteristics in large cohorts of individuals (6). The management of established ONJ lesions is complex and the clinical course is difficult to predict; prevention and the control of risk factors is thus very important. In consequence, one of the first strategic initiatives to prevent oral bisphosphonate-related ONJ in patients with osteoporosis should consist of ensuring the appropriate prescription of these drugs and, taking into account that this population has considerable requirements for dental treatment (including tooth extractions) (35), protocols for oral healthcare should be set in motion before starting treatment with an oral bisphosphonate.

In conclusion, in the present series, ONJ induced by oral bisphosphonates typically develops in women around 70 years of age, taking alendronate for 4.5 years, that underwent oral surgery in the 12 months prior. Most lesions are located in the posterior mandible and are classified as stage 2 at diagnosis. Although specific risk factors have been described, they are not detected in all patients, which lead us to speculate that there may be other, as yet unidentified risk factors. There are well-defined patterns of clinical presentation that can facilitate early diagnosis of ONJ.
